# Canagliflozin Ameliorates Myocardial Fibrosis and Cardiac Function in Chronic Heart Failure: A Dose‐Independent Therapeutic Approach

**DOI:** 10.1111/jcmm.70718

**Published:** 2025-08-10

**Authors:** Haomiao Yu, Zhenzhong Han, Wanpeng Chang, Huiming Zhou, Bingyu Du, Baoxue Jia, Hui Fu, Yanyan Yin, Mengfan Kan, Shaohong Yu, Zhongwen Zhang

**Affiliations:** ^1^ Department of Endocrinology and Metabology The First Affiliated Hospital of Shandong First Medical University & Shandong Provincial Qianfoshan Hospital Jinan China; ^2^ Department of Endocrinology and Metabology The Third Affiliated Hospital of Shandong First Medical University Jinan China; ^3^ Children's Hospital Affiliated to Shandong University & Jinan Children's Hospital Jinan China; ^4^ Cheeloo College of Medicine Shandong University Jinan China; ^5^ College of Medicine Shandong University of Traditional Chinese Medicine Jinan China

**Keywords:** Canagliflozin, chronic heart failure, fibrosis

## Abstract

The myocardial fibrosis leading to cardiac remodelling is a key factor in the progression of chronic heart failure. The present study aims to investigate the mechanism of canagliflozin's improvement of cardiac fibrosis in chronic heart failure rats and the effect of dose on efficacy. Chronic heart failure models were established by intraperitoneal injection of isoproterenol to rats for 10 days. The rats were then randomised into five groups: control rats, chronic heart failure rats, rats treated with a low dose of canagliflozin, rats treated with a high dose of canagliflozin and rats treated with enalapril. Canagliflozin was administered once daily for 4 weeks by gastric feeding. The rats were then euthanised after cardiac function analysis by echocardiography. Detection of chronic heart failure markers from serum was performed using enzyme‐linked immunosorbent assay kits. The tissue sections were examined by histological staining to assess the cardioprotective effect of canagliflozin in chronic heart failure rats. Myocardial interstitial fibrosis was evaluated by specific immunostaining. The control, chronic heart failure and low dose of canagliflozin groups were sequenced and analysed to identify differentially expressed genes, and the expression of selected genes was verified by qRT‐PCR. Echocardiographic measurements provided evidence supporting the ability of canagliflozin to improve cardiac function. Canagliflozin treatment reduced the chronic heart failure marker N‐terminal pro‐B‐type natriuretic peptide. HE staining and Masson's trichrome staining demonstrated that canagliflozin effectively reduced collagen deposition and alleviated myocardial fibrosis. The immunohistochemical staining revealed that treatment with canagliflozin inhibited the expression of fibrosis markers Collagen I, Collagen III and fibronectin 1. The differentially expressed genes identified through RNA sequencing were found to be enriched in the ECM–receptor interaction pathway. The results of qRT‐PCR demonstrated that canagliflozin reduced the level of differentially expressed genes involving collagen type I alpha 1 chain, collagen type I alpha 2 chain, collagen type III alpha 1 chain and fibronectin 1. Interestingly, the results did not show a trend toward better efficacy of high‐dose compared with low‐dose canagliflozin in the treatment of chronic heart failure. In summary, canagliflozin could directly improve myocardial fibrosis to control the progression of chronic heart failure. Canagliflozin may not be dose‐dependent in the treatment of chronic heart failure.

## 1 Introduction

Chronic heart failure (CHF) is a common fatal factor in cardiovascular diseases. The aging of the population has led to an increasing trend of both the incidence and prevalence of CHF [1]. Myocardial interstitial extracellular matrix (ECM) alterations play an important role in the development of myocardial remodelling in CHF. Diffuse accumulation of type I and type III collagen in the myocardial interstitium leads to myocardial fibrosis and decreased cardiac function [2]. The fibroblast proliferates and differentiates into myofibroblasts by forming contractile stress fibres and expressing α‐smooth muscle actin [3]. These processes result in myocardial fibrosis associated with reduced cardiac compliance and cardiac remodelling [4, 5]. Therefore, improved fibrosis and cardiac remodelling are needed to enhance cardiac function and thus slow down the progression of CHF.

Canagliflozin, an inhibitor of sodium‐glucose cotransporter 2 (SGLT‐2), has been shown to reduce the risk of worsening disease course in patients with CHF [6]. Canagliflozin can improve cardiac function in non‐diabetic myocardial infarction animals [7]. Canagliflozin has been shown to reduce myocardial glucose metabolism and oxidative stress and enhance myocardial fatty acid metabolism and ketone body cycling, thereby improving heart failure [8, 9]. In addition, a study has demonstrated that canagliflozin directly relaxes coronary arteries, improving blood flow in isolated mouse hearts [10]. A recent study has shown that canagliflozin may reduce the incidence or severity of cardiomyopathy and delay the aging process, acting as a treatment for age‐related heart disease [11]. Notably, the mechanisms by which canagliflozin directly improves myocardial remodelling remain unclear, and current speculation includes the fact that improvements in blood glucose control were comparatively minor and that improvements in diuresis, weight loss and antihypertension are insufficient to fully explain the observed differences [12, 13, 14]. Diabetic patients using high doses of canagliflozin demonstrate better glycaemic control, but in non‐diabetic CHF patients, high‐dose canagliflozin may affect their normal blood glucose levels. The use of relatively low doses of canagliflozin is necessary while ensuring cardiac efficacy. Therefore, the objective of this study was to examine whether canagliflozin treats CHF by directly improving myocardial remodelling and whether its therapeutic efficacy demonstrates dose dependency.

## 2 Materials and Methods

### 2.1 Experimental Animals

All animal experiments were approved by the Animal Experimentation Committee of the First Affiliated Hospital of Shandong First Medical University (Animal Ethical Number: 2020S014) and were conducted in accordance with the ARRIVE guidelines. Eight‐week‐old male SD rats, purchased from Beijing Weitong Lihua Laboratory Animal Technology Co. Ltd. and raised in the SPF animal room of the First Affiliated Hospital of Shandong First Medical University (Licence number: SCXK Beijing 2016‐0006), were placed under controlled conditions (12 h light/12 h dark cycle, 21°C–23°C, 40%–60% humidity) before the experiment, with access to water and food ad libitum to adapt to the new environment. Animals were randomly assigned prior to the experiment, and all tests and analyses were performed blindly by the experimenter. Euthanasia of animals was in accordance with AVMA approved guidelines.

### 2.2 Experimental Protocol

**FIGURE 1:**
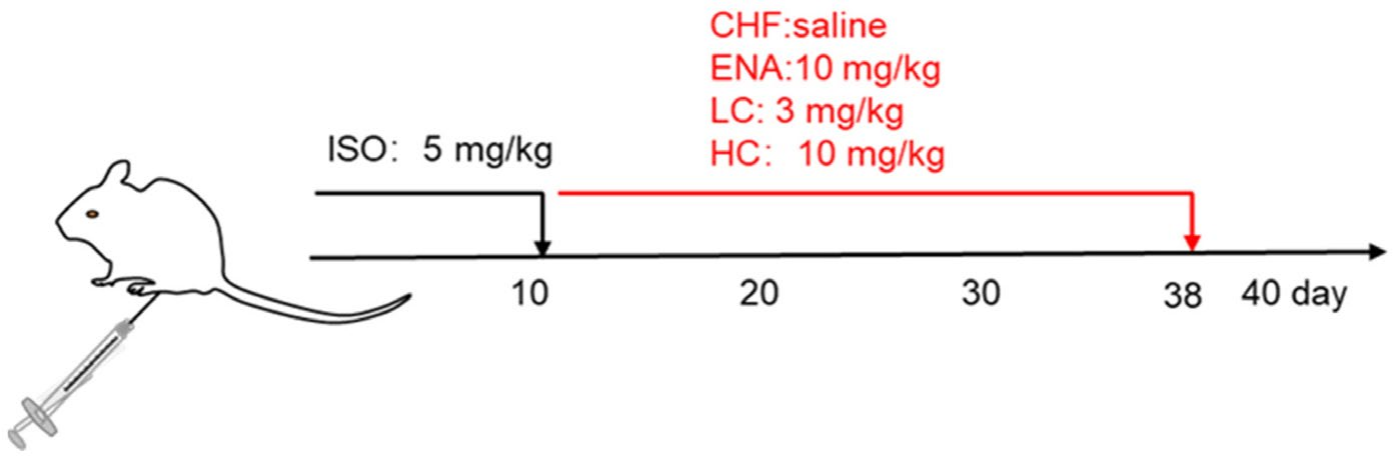
Schematic diagram of drug administration protocol in mice.

The rats were randomly divided into the control group (*n* = 8) and CHF group (*n* = 62). The isoproterenol (ISO)‐induced CHF rat models were established as described previously [15]. Rats were injected intraperitoneally with isoprenaline solution 5 mg/kg/d for 10 consecutive days. Regarding the study by HIRA et al. [16], Canagliflozin (Merck Serono, Beijing, China) was administered continuously by gavage for 4 weeks, and the drug dose was determined to be 3 and 10 mg/kg/d. After the model was established, the surviving 32 CHF rats were randomly divided into four groups: the CHF group (*n* = 8, oral administration of equal amounts of normal saline), the enalapril (ENA) group (*n* = 8, oral administration of enalapril 10 mg/kg/d), the low dose of canagliflozin (LC) group (*n* = 8, oral administration of canagliflozin 3 mg/kg/d) and the high dose of canagliflozin (HC) group (*n* = 8, oral administration of canagliflozin 10 mg/kg/d, Figure 1). The ENA group was included as a positive control to compare the efficacy of canagliflozin with a standard heart failure therapy, given the well‐documented benefits of ACE inhibitors in attenuating cardiac remodelling and fibrosis. After four weeks, rats were executed by anaesthesia with 2% sodium pentobarbital (40 mg/kg). Blood was taken from the femoral artery, centrifuged and serum collected and stored at −80°C for ELISA. Heart tissue from the middle of the left ventricle (2.5 mm above the apical region) was fixed with 4% paraformaldehyde and stained for histopathological analysis and immunohistochemical staining, and the rest of the left ventricular tissue was stored at −80°C for western blot assay and qRT‐PCR analysis.

### 2.3 Echocardiographic Assessment

Echocardiography was tested one day before ISO injection, one day after ISO injection and 4 weeks after drug intervention. Rats were anaesthetised with 2% sodium pentobarbital (40 mg/kg) and their chest hair was removed using an electric razor. The key parameters left ventricular ejection fraction (LVEF) and left ventricular shortening fraction (LVFS) were measured to evaluate cardiac function. The left ventricular end of systole volume (LVESV), left ventricular end of diastole volume (LVEDV), left ventricular internal diameter at end systole (LVIDs), left ventricular internal diameter at end‐diastole (LVIDd), left ventricular posterior wall diastole (LVPWd), left ventricular posterior wall systole (LVPWs), left ventricular anterior wall diastole (LVAWd) and left ventricular anterior wall systole (LVAWs) were measured from 2D‐guided M‐mode by software from Vevo3100 (VisualSonics Inc., Toronto, Ontario, Canada, Copyright licence has been obtained). The values of LVFS and LVEF were calculated using Vevo LAB software. Three measurement analyses were performed and the average of the three analyses was taken.

### 2.4 Histopathological Examination

The myocardial tissues were fixed with 4% paraformaldehyde for 48 h, and 4‐μm thick paraffin sections were prepared. These sections were stained with haematoxylin–eosin (HE) and Masson trichrome to assess histopathological changes and collagen deposition and then photographed at a magnification of 40×. Image‐Pro Plus (version 6.0, Copyright licence has been obtained) was used to calculate the collagen volume fraction (CVF): CVF (%) = collagen area/total area × 100%.

### 2.5 Immunohistochemistry

Hearts were fixed in 4% paraformaldehyde and embedded in paraffin. Heart sections were dewaxed and heated for antigen repair. The sections were treated with 3% hydrogen peroxide to inhibit the activity of endogenous peroxidase and then blocked with 4% bovine serum albumin. The sections were incubated overnight at 4°C using collagen I (1:150 dilution; Abcam, Cambridge, MA, USA), collagen III (1:700 dilution; Abcam) and FN1 (1:1000 dilution; Abcam) primary antibody. The slides were treated with a secondary antibody. Positive staining was detected with diaminobenzidine (DAB) and the sections were counterstained with haematoxylin. The images were observed under a 40× electron microscope and quantified using Image‐Pro Plus 6.0 software.

### 2.6 RNA Sequencing (RNA‐Seq)

Total RNA was extracted from the heart apical tissue using the RNeasy Mini Kit (250) Qiagen#74106 kit. The three biological repeats of the Control, CHF and LC groups were used for RNA quality control and quantification, respectively. Strand‐specific libraries were prepared after ribosomal RNA depletion and sequenced on a NovaSeq 6000 instrument using a paired‐end sequencing chemistry. The raw off‐machine data were first processed to obtain high‐quality sequences, and then the high‐quality sequences were compared with reference genes and the results are quantified for transcriptome expression. Differentially expressed genes were calculated and differentially screened by Fragments Per Kilobase per Million (FPKM), etc. Screening criteria: *p* value < 0.05 and the fold change (FC) is 2 times up (FC ≥ 2) or 2 times down (FC ≤ 2) and eliminating the differentially expressed genes with FPKM < 1 in each group. The visualisations sequencing data are created using the R programming language and integrated into the web‐based bioinformatics tools (http://www.bioinformatics.com.cn). Functional enrichment analysis of differentially expressed genes was carried out using the STRING 11.5 database [17] (https://cn.string‐db.org). After hiding the unconnected nodes, the protein–protein interaction (PPI) network was obtained. The list of PPI pairs was downloaded for further analysis and visualised by the Molecular Complex Detection (MCODE) [18] tool of Cytoscape 3.7.1 [19] (http://cytoscape.org/). Gene Ontology (GO) functional analysis and Kyoto Encyclopedia of Genes and Genomes (KEGG) enrichment analysis were performed using DAVID [20] (https://david.ncifcrf.gov/) and Metascape [21] (https://metascape.org/). Data visualisation was performed using bioinformatics online tools and Cytoscape 3.7.1.

### 2.7 Quantitative Reverse Transcription‐PCR Analysis

Real‐time PCR was operated (LightCycler 480 Thermocycler; Roche Applied Science, Mannheim, Germany) using a SYBR Green qPCR Kit (Toyobo) according to an optimised protocol and using optimised primer sets. Total RNA was extracted using TRIZOL Reagent (Genstar, Beijing, China) and was reversed transcribed using a cDNA reverse transcription kit (Accurate Biology, Hunan, China). The routine was set as follows: cDNA denaturation at 95°C for 2 min and 30 s, 35 cycles of 94°C for 14 s, 57°C for 15 s and 70°C for 30 s. The products of PCR were run in 1% agarose gel electrophoresis. Quantitative real‐time PCR was used to determine the mRNA levels of collagen 1 alpha 1 (COL1A1, Forward: 5′‐GAGGGCCAAGACGAAGACATC‐3′; Reverse: 5′‐CAGATCACGTCATCGCACAAC‐3′), collagen 1 alpha 2 (COL1A2, Forward: 5′‐CAGGTGGAACAGCGGTTCTAC‐3′; Reverse: 5′‐TGGGACCTTGTCCCTGAAG‐3′), collagen 3 alpha 1 (COL3A1, Forward: 5′‐TGGTGGTTTTCAGTTCAGCCT‐3′; Reverse: 5′‐GCAGCCATCCATCTCTTCCT‐3′) and fibronectin 1 (FN1, Forward: 5′‐CAGTGGGAGACCTCGAGAAG‐3′; Reverse: 5′‐TGGGTTGGTGTAGGTGTGAG‐3′). Glyceraldehyde‐3‐Phosphate Dehydrogenase, internal control (GAPDH: Forward: 5′‐TGAACGGGAAGCTCACTGG‐3′; Reverse: 5′‐TCCACCACCCTGTTGCTGTA‐3′) Relative gene expression was calculated based on 2^−ΔΔct^ method using β‐Actin as an internal control.

### 2.8 Enzyme‐Linked Immunosorbent Assay (ELISA) Analysis

Blood samples were collected from rats after 4 weeks of intervention to prepare serum specimens. N‐terminal pro‐B‐type natriuretic peptide (NT‐pro BNP) (Mlbio, Shanghai, China) was measured from serum using ELISA Kits according to the manufacturer instructions.

### 2.9 Statistical Analysis

Data were analysed using GraphPad Prism 8 software (GraphPad, SanDiego, CA, USA, Copyright licence has been obtained) and the results are presented as mean standard error of the mean (SEM). For longitudinal comparisons across multiple time points, analysis was performed using repeated measures ANOVA, followed by Bonferroni‐corrected pairwise comparisons. *p* < 0.05 was considered statistically significant.

## 3 Results

### 3.1 The Effects of Low‐Dose and High‐Dose Canagliflozin on ISO‐Induced Cardiac Dysfunction Were Similar

**FIGURE 2:**
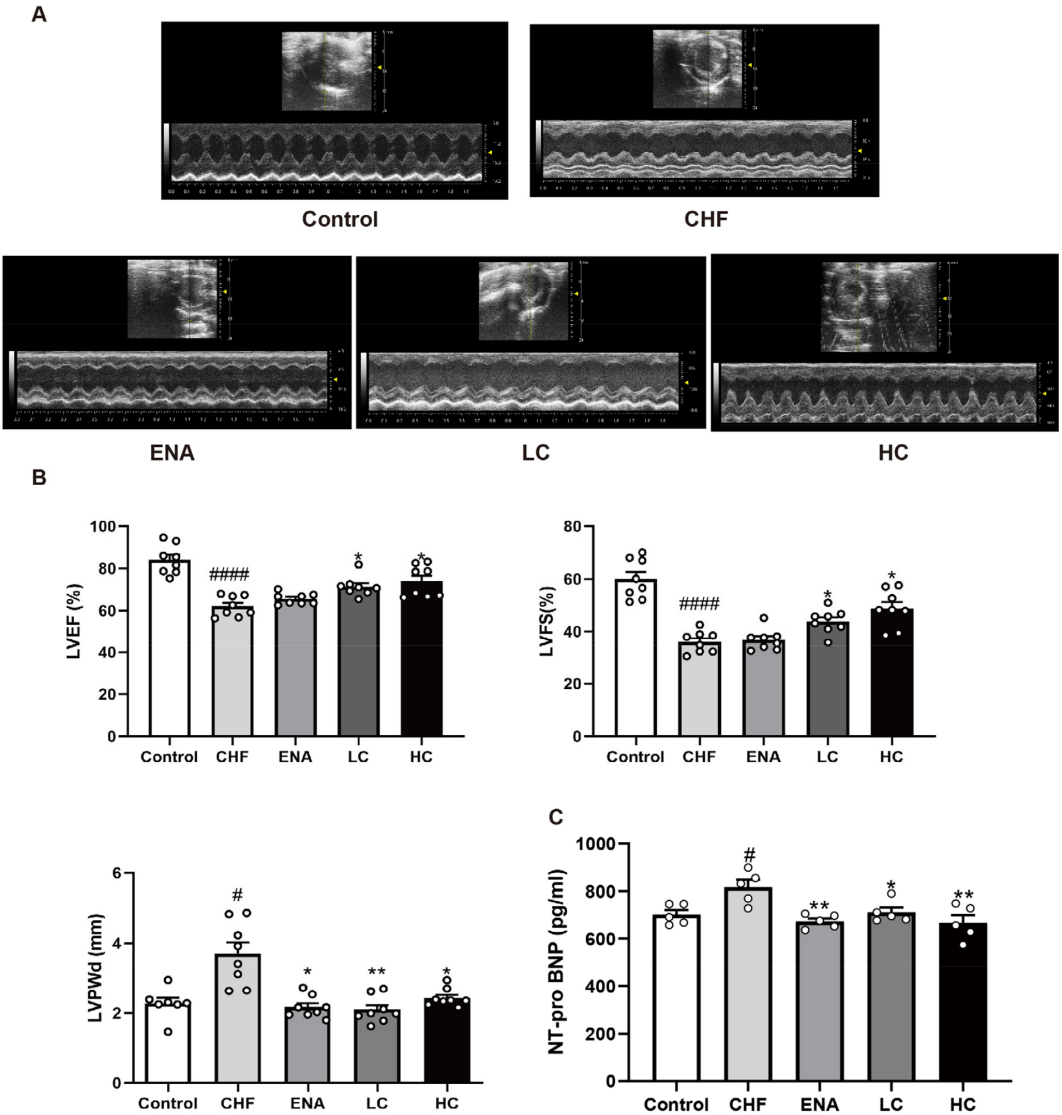
Effect of canagliflozin on ISO‐induced echocardiographic parameters in rats. (A) Representative images of echocardiography exhibiting changes in cardiac function in each group. (B) Quantitative data of echocardiographic measurements. (C) The changes in serum contents of NT‐pro BNP. Data are shown as the mean ± SEM, *n* = 4–8 per group. Control, control group; CHF, chronic heart failure group; ENA, enalapril 10 mg/kg/d group; LC, canagliflozin 3 mg/kg/d group; HC, canagliflozin 10 mg/kg/d group. LVEF, left ventricular ejection fraction; LVFS, left ventricular fractional shortening; LVPWd, left ventricular posterior wall thickness at end‐diastole; NT‐pro BNP, N‐terminal pro brain natriuretic peptide. #*p* < 0.05 vs. Control group; ####*p* < 0.0001 vs. Control group; *p < 0.05 vs. CHF group; **p < 0.01 vs. CHF group.

Application of echocardiography to evaluate the efficacy of canagliflozin in the treatment of CHF. After 4 weeks of drug intervention, the LVEF and LVFS of rats in the CHF group (LVEF, *p* < 0.0001; LVFS, *p* < 0.0001) were significantly lower than those in the control group, indicating that the CHF rat model was successfully established. Notably, pre‐treatment LVEF and LVFS values in all groups were comparable (all *p* > 0.05), indicating baseline homogeneity (Tables 2 and 3). LVEF and LVFS levels were significantly higher in the LC group (LVEF, *p* < 0.05; LVFS, *p* < 0.05) and HC group (LVEF, *p* < 0.05; LVFS, *p* < 0.05). Compared with the Control group, the levels of LVPWd in the CHF group (*p* < 0.05) were significantly higher, suggesting the presence of myocardial hypertrophy in the rats. In contrast, LVPWd levels were significantly lower in the LC group (*p* < 0.01) and HC group (*p* < 0.05) (Figure 2A,B; Table 1). Serum NT‐Pro BNP levels were further measured to confirm the protective effect of canagliflozin on CHF. Compared to the CHF group, the NT‐pro BNP levels were significantly lower in the ENA group (*p* < 0.01), the LC group (*p* < 0.05) and the HC group (*p* < 0.01) (Figure 2C).

**TABLE 1:** Canagliflozin improved left heart function in rats.

Group	Control	CHF	ENA	LC	HC
LVEF (%)	84.11 ± 2.47	62.03 ± 1.72^####^	65.54 ± 0.93	71.38 ± 1.67*	73.93 ± 2.64*
LVFS (%)	60.00 ± 2.63	35.94 ± 1.48^####^	36.70 ± 1.44	43.86 ± 1.61*	48.70 ± 2.52*
LVPWd (mm)	2.27 ± 0.16	3.70 ± 0.32^#^	2.16 ± 0.11*	2.09 ± 0.13**	2.43 ± 0.09*
LVPWs (mm)	3.78 ± 0.29	3.97 ± 0.26	3.00 ± 0.19*	3.34 ± 0.18	3.77 ± 0.24
LVAWd (mm)	2.60 ± 0.22	2.35 ± 0.18	2.34 ± 0.14	2.17 ± 0.15	2.12 ± 0.17
LVAWs (mm)	4.04 ± 0.08	3.10 ± 0.23^#^	3.30 ± 0.11	2.99 ± 0.10	3.16 ± 0.17
LVIDd (mm)	6.47 ± 0.31	6.64 ± 0.18	6.64 ± 0.32	7.16 ± 0.24	6.55 ± 0.28
LVIDs (mm)	2.93 ± 0.31	4.16 ± 0.20^#^	4.18 ± 0.25	3.92 ± 0.23	3.61 ± 0.28
LVEDV (μl)	218.70 ± 24.06	230.50 ± 13.62	233.04 ± 26.20	246.68 ± 22.68	209.77 ± 20.02
LVESV (μl)	37.19 ± 8.98	81.00 ± 9.20^#^	81.39 ± 13.24	63.47 ± 9.05	60.51 ± 10.65

*Note:* The results are expressed as mean ± SEM, *n* = 4–8 per group.

Abbreviations: Control, control group; CHF, chronic heart failure group; ENA, enalapril 10 mg/kg/d group; HC, canagliflozin 10 mg/kg/d group; LC, canagliflozin 3 mg/kg/d group; LVEF, left ventricular ejection fraction; LVFS, left ventricular fractional shortening; LVPWd, left ventricular posterior wall thickness at end‐diastole; LVPWs, left ventricular posterior wall thickness at end systole; LVAWd, left ventricular anterior wall diastole; LVAWs, left ventricular end‐systolic anterior wall thickness; LVIDd, left ventricular end‐diastolic inner‐dimension; LVIDs, left ventricular end‐systolic inner‐dimension; LVEDV, left ventricular end‐diastolic volume; LVESV, left ventricular end‐systolic volume.

* *p* < 0.05 vs. ISO group.

** *p* < 0.01 vs. ISO group.

^#^ *p* < 0.05 vs. control group.

^####^ *p* < 0.0001 vs. control group.

**TABLE 2:** Changes in LVEF values of five mouse groups across pre‐modelling, post‐modelling and post‐treatment phases.

Group	Control	CHF	ENA	LC	HC
Control	85.74 ± 2.80	93.54 ± 1.54	79.63 ± 4.78	86.78 ± 3.83	87.37 ± 2,29
Model	87.70 ± 2.11	65.43 ± 1.84^####^	65.34 ± 1.04^####^	67.90 ± 1.77^####^	59.07 ± 3.33^####^
Treatment	84.11 ± 2.47	62.03 ± 1.72^####^	65.54 ± 0.93*	71.38 ± 1.67*	73.93 ± 2.64*

*Note:* The results are expressed as mean ± SEM, *n* = 4–8 per group.

Abbreviations: Control, control group; CHF, chronic heart failure group; ENA, enalapril 10 mg/kg/d group; HC, canagliflozin 10 mg/kg/d group; LC, canagliflozin 3 mg/kg/d group; LVEF, left ventricular ejection fraction.

* *p* < 0.05 vs. ISO group.

^####^ *p* < 0.0001 vs. control group.

**TABLE 3:** Changes in LVFS values of five mouse groups across pre‐modelling, post‐modelling and post‐treatment phases.

Group	Control	CHF	ENA	LC	HC
Control	57.20 ± 3.42	62.71 ± 3.73	49.25 ± 4.86	59.97 ± 4.74	58.96 ± 2.74
Model	59.50 ± 2.95	37.41 ± 1.68^###^	36.05 ± 1.32^###^	39.75 ± 1.77^###^	36.68 ± 3.14^###^
Treatment	60.00 ± 2.63	35.94 ± 1.48^####^	36.70 ± 1.44	43.86 ± 1.61*	48.70 ± 2.52*

*Note:* The results are expressed as mean ± SEM, *n* = 4–8 per group.

Abbreviations: Control, control group; CHF, chronic heart failure group; ENA, enalapril 10 mg/kg/d group; HC, canagliflozin 10 mg/kg/d group; LC, canagliflozin 3 mg/kg/d group; LVFS, left ventricular fractional shortening.

* *p* < 0.05 vs. ISO group.

^###^ *p* < 0.001 vs. control group.

^####^ *p* < 0.0001 vs. control group.

These results suggested progressive myocardial hypertrophy and decreased cardiac function after ISO infusion. In contrast, canagliflozin improved ISO‐induced cardiac insufficiency and myocardial hypertrophy by enhancing myocardial contractility. No significant differences were observed among the three drug administration conditions, and cardiac function did not improve more significantly with increasing doses of canagliflozin.

### 3.2 Canagliflozin Improved ISO‐Induced Cardiac Remodelling

The effects of canagliflozin on ISO‐induced cardiac pathomorphology and collagen fibre deposition in rats were observed using HE and Masson trichrome staining.

**FIGURE 3:**
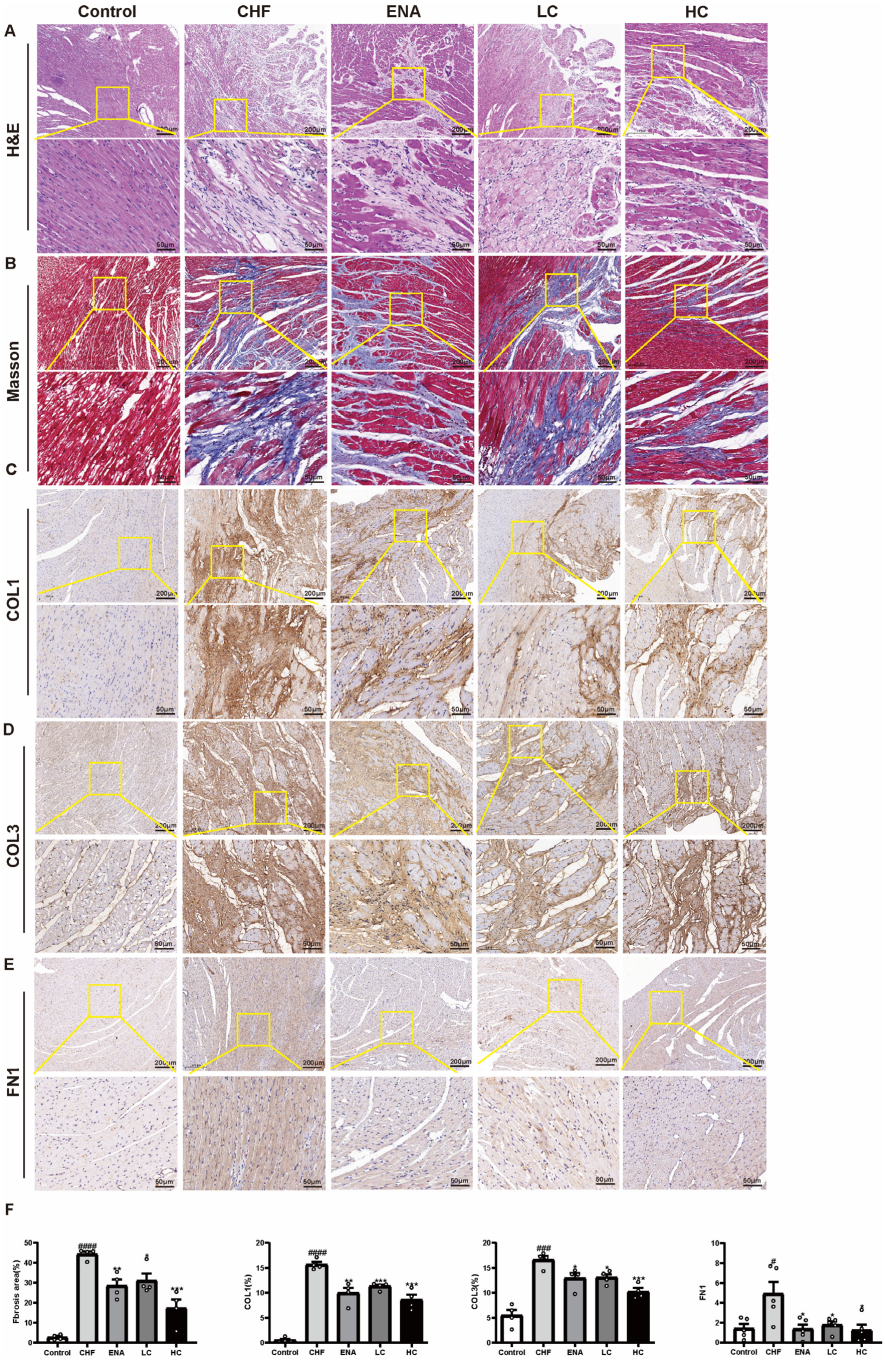
Effects of canagliflozin on cardiac fibrosis and remodelling in rats. (A) Representative images of HE staining of heart tissues from different groups (Microscope magnification 10×, scale bar indicates 200 μm; Microscope magnification 40×, scale bar indicates 50 μm). (B) Representative images of Masson trichrome staining of different groups of heart tissue (Microscope magnification 10×, scale bar indicates 200 μm; Microscope magnification 40×, scale bar indicates 50 μm). (C‐E) Immunohistochemical analysis of collagen I, collagen III and FN1 in different groups of heart sections (Microscope magnification 10×, scale bar indicates 200 μm; Microscope magnification 40×, scale bar indicates 50 μm). (F) Quantitative analysis of collagen deposition and percentage of type I, type III collagen and FN1 area. Data are shown as the mean ± SEM, *n* = 4–5 per group. #*p* < 0.05 vs. Control group; ###*p* < 0.001 vs. Control group; ####*p* < 0.0001 vs. Control group; *p < 0.05 vs. CHF group; **p < 0.01 vs. CHF group; ***p < 0.001 vs. CHF group. Control, control group; CHF, chronic heart failure group; COL1, Collagen I; COL3, Collagen III; ENA, enalapril 10 mg/kg/d group; HC, canagliflozin 10 mg/kg/d group; HE, haematoxylin–eosin; LC, canagliflozin 3 mg/kg/d group.

HE staining showed that cardiac myocytes in the CHF group were obviously sparse and hypertrophied, and myocardial fibres were partially broken and disorganised. Myocardial fibre alignment was improved in the ENA, LC and HC groups of rats (Figure 3A). Masson's trichrome staining showed significantly higher collagen fibre deposition in the CHF group (*p* < 0.0001) compared to the control group. The deposition of collagen fibres was significantly reduced in the ENA group (*p* < 0.01), the LC group (*p* < 0.05) and the HC group (*p* < 0.001) compared to the CHF group (Figure 3B,F).

The expression of myocardial fibrosis markers COL1, COL3 and FN1 was detected by immunohistochemistry to investigate the ameliorative effect of canagliflozin on myocardial fibrosis. Quantitative image analysis showed a significant increase in the expression of COL1 (*p* < 0.0001), COL3 (*p* < 0.001) and FN1 (*p* < 0.05) in the CHF group compared to the control group. The expression of COL1, COL3 and FN1 was significantly reduced in the ENA group (COL1, *p* < 0.01; COL3, *p* < 0.05; FN1, *p* < 0.05), the LC group (COL1, *p* < 0.001; COL3, *p* < 0.05; FN1, *p* < 0.05) and the HC group (COL1, *p* < 0.001; COL3, *p* < 0.001; FN1, *p* < 0.05) compared to the CHF group. In addition, the expression of COL1, COL3 and FN1 in rats in the HC group was lower than that in the ENA and LC groups, but there was no statistical difference (Figure 3C–F). These results suggested that canagliflozin can reduce myocardial fibrosis and improve cardiac function.

### 3.3 Canagliflozin Exerted Cardiac Protective Effects Through the ECM–Receptor Interaction Pathway

**FIGURE 4:**
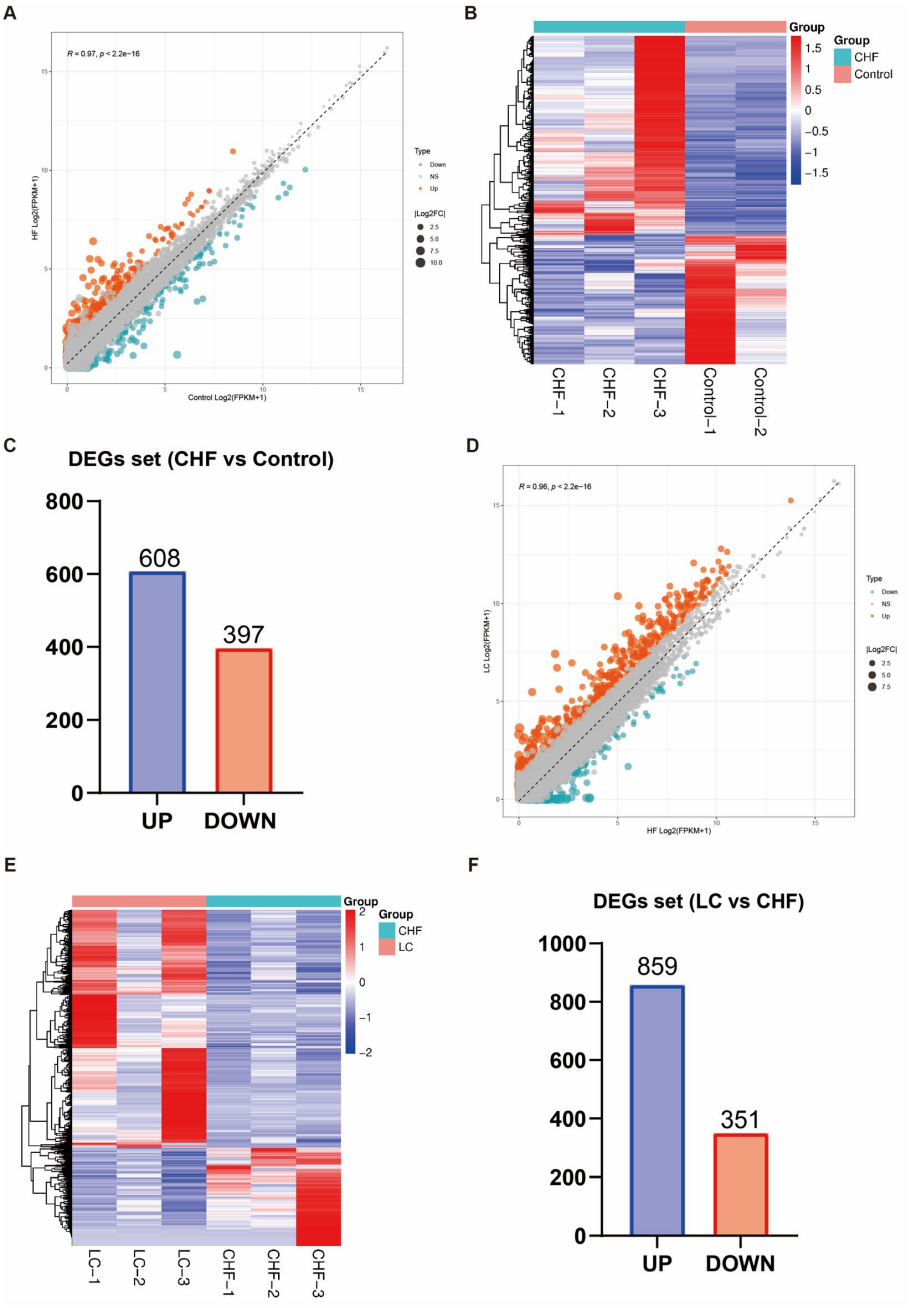
RNA‐seq was used to detect genome‐wide transcripts and identify differentially expressed genes. (A) Volcano plot of 18,713 genes identified through RNA‐seq. The abscissa is log_2_ (fold change), and the vertical coordinate is the negative logarithm of the value of Q with a base of 10, that is −log10 (Q). The larger the value, the more significant the difference. (B) Heat map of differential genes. Each row represents a differential gene and each column represents the same rat sample, with 2 replicates in the Control group and 3 replicates in the CHF group. (C) Histogram of differential genes. Control, control group; CHF, chronic heart failure group. (D) Volcano plot of 19,143 genes identified through RNA‐seq. The abscissa is log_2_ (fold change), and the vertical coordinate is the negative logarithm of the value of Q with a base of 10, that is −log_10_ (Q). The larger the value, the more significant the difference. (E) Heat map of differential genes. Each row represents a differential gene, each column represents the same rat sample, and each group has 3 replicates. (F) Histogram of differential genes. CHF, chronic heart failure group; LC, canagliflozin 3 mg/kg/d group.

The whole transcriptome was used to study the effect of canagliflozin on gene transcription in CHF rats, and the sequencing data were verified by qRT‐PCR. RNA‐seq detected 18,713 genes in the apical tissues of the Control and CHF groups, of which 1005 differentially expressed genes, including 608 upregulated and 397 downregulated genes. Then 19,143 genes were detected in the apical tissues of the CHF and LC groups, of which 1210 were differentially expressed, including 859 upregulated genes and 351 downregulated genes (Figure 4).

**FIGURE 5:**
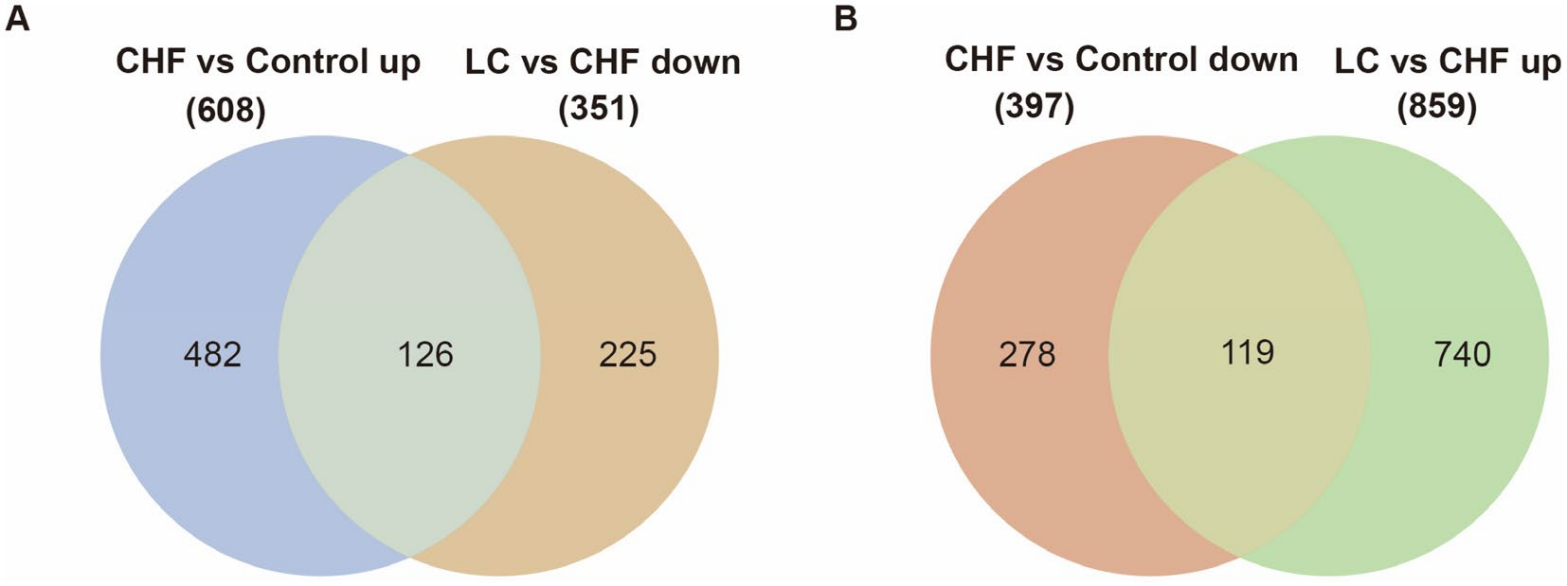
Venn diagram showing differentially expressed genes for up‐ versus downregulation of CHF by canagliflozin. (A) The 608 upregulated genes obtained from the comparison of Control and CHF groups were intersected with the 351 downregulated genes obtained from the comparison of LC and CHF groups, and the 126 differential genes obtained for the downregulation of heart failure by canagliflozin. (B) The 397 downregulated genes obtained from the comparison of the Control and CHF groups were intersected with the 859 upregulated genes obtained from the comparison of the LC and CHF groups to obtain 119 differential genes for upregulation of heart failure by canagliflozin. Control, control group; CHF, chronic heart failure group; LC, canagliflozin 3 mg/kg/d group.

**FIGURE 6:**
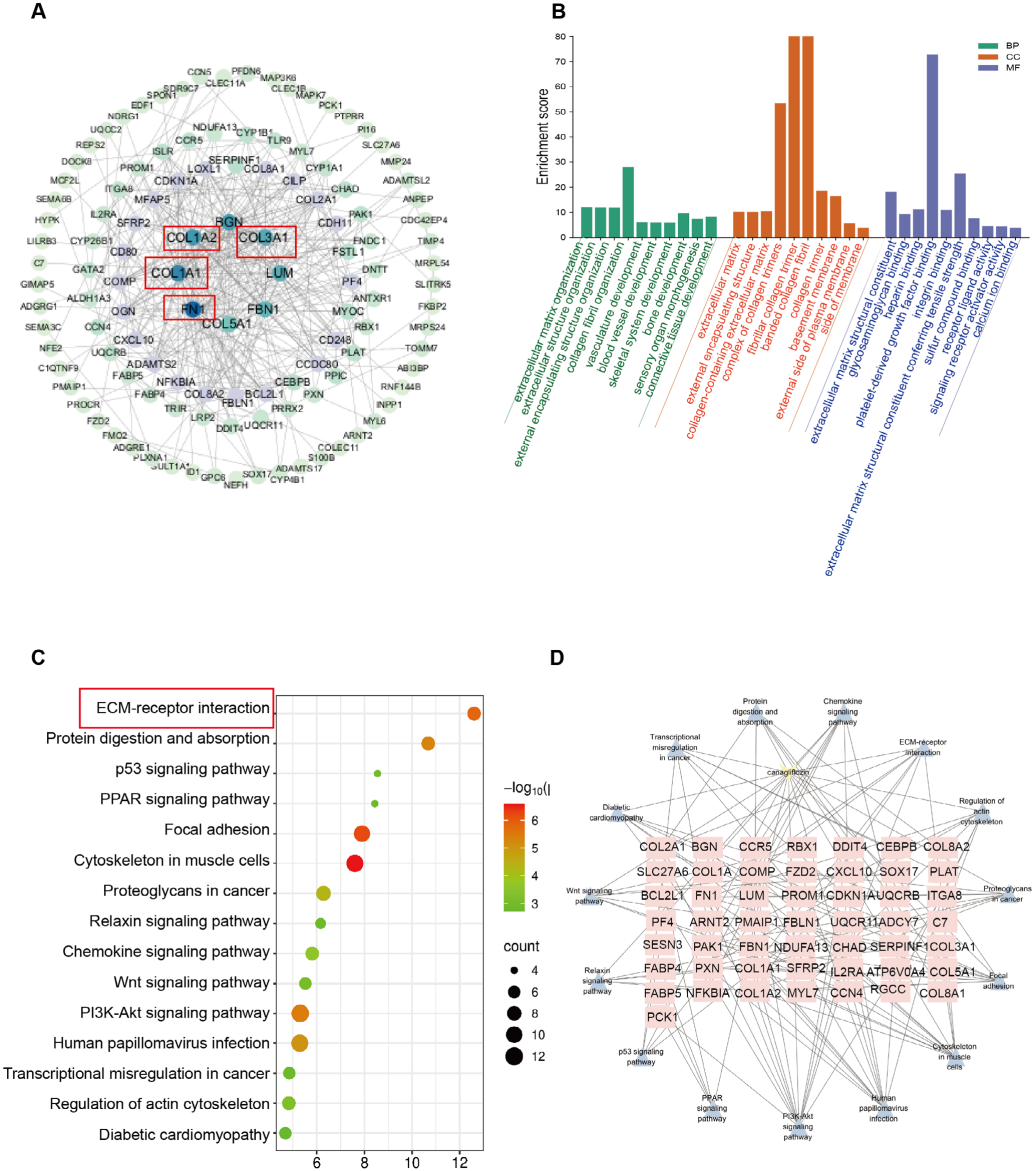
Enrichment analysis of the targets of canagliflozin in treating CHF. (A) PPI network. The size and colour of the nodes are displayed in descending order of degree values from large to small and from blue to green. (B) The GO analysis includes biological process, cellular component and molecular function, respectively. (C) KEGG pathway enrichment analysis. The bubble size represents the number of the target in the enriched pathway terms, bubble colour represents the pathway's q value. (D) Target‐pathway network. The 50 square nodes in the middle represent the targets on the pathway, and the 15 blue nodes represent the pathways. PPI, protein–protein interaction; GO, Gene Ontology; KEGG, Kyoto Encyclopedia of Genes and Genomes.

The 126 differential genes for downregulated CHF and 119 differential genes for upregulated CHF were obtained by taking the intersection of differential genes between the control group, CHF group and LC group (Figure 5). The 245 differentially expressed genes were arranged in descending order of degree in concentric circles to illustrate the potential relationships between targets revealed by the PPI network (Figure 6A). From the PPI network, an MCODE module consisting of 5 targets was identified, which may play a more important regulatory role.

GO analysis described the biological process, molecular function and cellular component of differentially expressed genes. In terms of biological process, the differential genes were mainly concentrated in extracellular matrix organisation and collagen fibril organisation. The cellular components were mainly concentrated in collagen‐containing extracellular matrix and the complex of collagen trimers. For molecular functions, the differential genes were mainly related to extracellular matrix structural constituent (Figure 6B). The genes were enriched in the ECM–receptor interaction, diabetic cardiomyopathy, chemokine signalling pathway, protein digestion and absorption, and other signalling pathways according to the KEGG enrichment analysis (Figure 6C).

**FIGURE 7:**
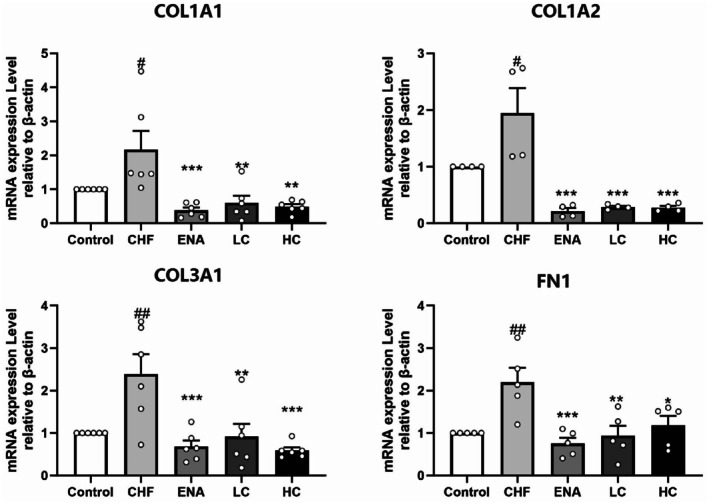
Effect of canagliflozin on indicators of fibrosis. The gene expression levels of COL1A1, COL1A2, COL3A1 and FN1 were detected by qRT‐PCR. The internal reference was β‐Actin. Data are shown as the mean ± SEM, *n* = 4–8 per group. #*p* < 0.05 vs. Control group; ##*p* < 0.01 vs. Control group; ####*p* < 0.0001 vs. Control group; *p < 0.05 vs. CHF group; **p < 0.01 vs. CHF group; ***p < 0.001 vs. CHF group. Control, control group; CHF, chronic heart failure group; ENA, enalapril 10 mg/kg/d group; LC, canagliflozin 3 mg/kg/d group; HC, canagliflozin 10 mg/kg/d group.

Based on the results of KEGG pathway enrichment analysis, a targeting pathway network was constructed using Cytoscape 3.7.1 (Figure 6D). COL1A1, COL1A2, COL3A1 and FN1 in the protein functional module were enriched in the ECM–receptor interaction pathway, so these four targets needed to be further validated by PCR. Compared with the Control group, the mRNA levels of COL1A1 (*p* < 0.05), COL1A2 (*p* < 0.05), COL3A1 (*p* < 0.01) and FN1 (*p* < 0.01) in the CHF group were significantly upregulated. The mRNA levels of COL1A1, COL1A2, COL3A1, and FN1 were significantly reduced by ENA (COL1A1, *p* < 0.001; COL1A2, *p* < 0.001; COL3A1, *p* < 0.001; FN1, *p* < 0.001), LC (COL1A1, *p* < 0.01; COL1A2, *p* < 0.001; COL3A1, *p* < 0.01; FN1, *p* < 0.01) and HC (COL1A1, *p* < 0.01; COL1A2, *p* < 0.001; COL3A1, *p* < 0.001; FN1, *p* < 0.05) treatment (Figure 7). The above results suggested that canagliflozin can reduce the mRNA expression levels of these differential genes.

### 3.4 Discussion

CHF is characterised by progressive left ventricular dysfunction and reduced ejection fraction, usually associated with ventricular remodelling due to myocardial fibrosis [22, 23, 24, 25, 26]. The main features of myocardial fibrosis are fibroblast proliferation, excessive collagen synthesis and extracellular matrix deposition further reducing myocardial compliance [27, 28, 29]. We constructed an animal model of CHF using ISO and modelled successful rats with cardiac pathology and structural heart alterations comparable to those seen in human heart failure [30]. In the present study, we demonstrated that oral administration of canagliflozin protected the myocardium and improved CHF in non‐diabetic rats. However, our results showed no dose dependence of canagliflozin for CHF.

NT‐proBNP might be a better diagnostic biomarker of CHF than BNP to indicate the severity of heart disease [31, 32, 33]. The reduction of NT‐proBNP level predicts an improvement in clinical symptoms, and there is a positive correlation between the risk of death and the assessment of NT‐proBNP [34]. This was reflected in our results, where the beneficial effects of canagliflozin on heart failure were shown in the reduction of heart failure marker NT‐pro BNP. HE and Masson's staining showed a significant increase in the volume of collagen fibres in the CHF group, as well as an increase in the expression of COL1, COL3 and FN1 in the CHF group in immunohistochemistry. However, canagliflozin treatment decreased the collagen fibre volume and expression of COL1 and COL3 as well as FN1. Our findings showed that canagliflozin may improve CHF by reducing myocardial fibrosis.

It has been suggested that the beneficial effects of canagliflozin on CHF may be through lowering blood glucose, renoprotective effects, lowering circulating blood pressure and body weight [35, 36, 37, 38], and there are also relevant studies showing that long‐term administration of canagliflozin reduces the size of myocardial infarction in rats independent of blood glucose status [7]. It has been shown that SGLT2 receptor inhibitors had beneficial effects on the heart by acting directly on the sodium‐hydrogen exchanger in the heart and reducing the sodium concentration in the cytoplasm of cardiomyocytes [10], and it has also been shown that inhibition of SGLT1 receptors in the heart can have a protective effect against heart failure due to myocardial infarction [39, 40]. In addition, our sequencing results showed that the improvement of CHF by canagliflozin may be related to ECM–receptor interaction pathway, which controls cell activity such as adhesion, migration, differentiation, proliferation and apoptosis. Overexpression of COL1A1 and COL1A2 in the ECM–receptor interaction pathway led to myocardial fibrosis, as demonstrated in the CHF group of our experiment. COL1A1 and COL1A2 mRNA expression levels were reduced after canagliflozin treatment.

Previous studies have shown that preventing and improving the expansion of the ECM caused by excess collagen remains an important component of the treatment of CHF [41]. The reason was that the extracellular matrix expansion of excess collagen and its adverse effects were reversible. Specific activation of myocardial fibroblasts in mice leads to excessive collagen accumulation and myocardial fibrosis and results in a heart failure phenotype [42]. Myocardial fibrosis occurs when collagen homeostasis is dysregulated and excess collagen accumulates in the interstitial matrix secreted by myofibroblasts. The conversion of fibroblasts to myofibroblasts involves the expression of α‐smooth muscle actin, which is unique to smooth muscle cells [43]. The composition of the ECM includes fibronectin, and FN1 expression in fibroblasts is increased in the presence of myocardial fibrosis and myocardial failure [44]. In agreement with the results of these studies, we found that canagliflozin significantly reduced the mRNA level of COL3A1 and FN1.

A meta‐analysis showed that the clinical effects of the lowest commercially available dose of SGLT2 inhibitors on glycated haemoglobin and body weight were similar to those of higher doses [45]. In an EMPA‐REG Outcomes trial of SGLT2 inhibitor, the two tested doses of empagliflozin had the same effect on cardiac outcomes [46]. The use of far lower clinical doses of engramine was as effective as standard doses of telmisartan in improving cardiac fibrosis [47]. This is similar to our results, where low‐dose versus high‐dose canagliflozin improved myocardial fibrosis as well as standard‐dose enalapril.

In the present study, we demonstrated that canagliflozin, an SGLT2 inhibitor, significantly improved myocardial fibrosis and cardiac function in a non‐diabetic CHF model. However, no dose‐dependent differences were observed between the low‐dose (3 mg/kg/d) and high‐dose (10 mg/kg/d) canagliflozin treatment groups. This finding is consistent with previous studies suggesting that the therapeutic effects of SGLT2 inhibitors may not strictly correlate with dose escalation. Several factors may explain the lack of dose‐dependent differences in our experiments. First, the pharmacological effects of canagliflozin, particularly its cardioprotective properties, may reach a plateau at relatively low doses. Studies have shown that SGLT2 inhibitors exert their beneficial effects on the heart through mechanisms independent of glucose‐lowering, such as modulation of sodium‐hydrogen exchange, reduction of oxidative stress and improvement of myocardial energetics. These mechanisms may be maximally activated at lower doses, rendering higher doses unnecessary for additional benefits. Second, the dose range selected in our study (3 mg/kg/d to 10 mg/kg/d) might have been within the therapeutic window where the drug's efficacy is already optimal. Previous clinical and preclinical studies have reported similar outcomes with low and high doses of SGLT2 inhibitors. For instance, in the EMPA‐REG OUTCOME trial, both low and high doses of empagliflozin demonstrated comparable cardiovascular benefits, suggesting a ceiling effect in their therapeutic actions.

## 4 Conclusions

Taken together, our study demonstrated that canagliflozin improves myocardial fibrosis and cardiac function in CHF rats, and the efficacy is not dose dependent. This study may stimulate clinical studies to implement lower doses of canagliflozin without affecting the efficacy of the current use of standard doses of canagliflozin in patients with CHF.

## Author Contributions

**Haomiao Yu:** data curation (equal). **Zhenzhong Han:** conceptualization (equal). **Wanpeng Chang:** data curation (equal). **Huiming Zhou:** software (equal). **Bingyu Du:** data curation (equal). **Baoxue Jia:** formal analysis (equal). **Hui Fu:** formal analysis (equal). **Yanyan Yin:** data curation (equal). **Mengfan Kan:** writing – original draft (equal). **Shaohong Yu:** formal analysis (equal). **Zhongwen Zhang:** conceptualization (equal).

## Conflicts of Interest

The authors declare no conflicts of interest.

## Data Availability

The data that support the findings of this study are available from the corresponding author upon reasonable request.
